# VCAM-1^+^ placenta chorionic villi-derived mesenchymal stem cells display potent pro-angiogenic activity

**DOI:** 10.1186/s13287-016-0297-0

**Published:** 2016-04-04

**Authors:** Wenjing Du, Xue Li, Ying Chi, Fengxia Ma, Zongjin Li, Shaoguang Yang, Baoquan Song, Junjie Cui, Tao Ma, Juanjuan Li, Jianjian Tian, Zhouxin Yang, Xiaoming Feng, Fang Chen, Shihong Lu, Lu Liang, Zhi-Bo Han, Zhong-Chao Han

**Affiliations:** The State Key Laboratory of Experimental Hematology, Institute of Hematology and Hospital of Blood Diseases, Chinese Academy of Medical Sciences & Peking Union Medical College, No.288, Nanjing Road, Heping District, Tianjin, 300020 China; Beijing Institute of Health and Stem Cells, No.1 Kangding Road, BDA, Beijing, 100176 China; National Engineering Research Center of Cell Products, No.80, Fourth Avenue, TEDA, Tianjin, 300457 China

**Keywords:** Mesenchymal stem cells, Placenta, Angiogenesis, Paracrine, Vascular cell adhesion molecule-1 (CD106)

## Abstract

**Introduction:**

Mesenchymal stem cells (MSCs) represent a heterogeneous cell population that is promising for regenerative medicine. The present study was designed to assess whether VCAM-1 can be used as a marker of MSC subpopulation with superior angiogenic potential.

**Methods:**

MSCs were isolated from placenta chorionic villi (CV). The VCAM-1^+/−^ CV-MSCs population were separated by Flow Cytometry and subjected to a comparative analysis for their angiogenic properties including angiogenic genes expression, vasculo-angiogenic abilities on Matrigel *in vitro and in vivo*, angiogenic paracrine activities, cytokine array, and therapeutic angiogenesis in vascular ischemic diseases.

**Results:**

Angiogenic genes, including HGF, ANG, IL8, IL6, VEGF-A, TGFβ, MMP_2_ and bFGF, were up-regulated in VCAM-1^+^CV-MSCs. Consistently, angiogenic cytokines especially HGF, IL8, angiogenin, angiopoitin-2, μPAR, CXCL1, IL-1β, IL-1α, CSF2, CSF3, MCP-3, CTACK, and OPG were found to be significantly increased in VCAM-1^+^ CV-MSCs. Moreover, VCAM-1^+^CV-MSCs showed remarkable vasculo-angiogenic abilities by angiogenesis analysis with Matrigel *in vitro and in vivo* and the conditioned medium of VCAM-1^+^ CV-MSCs exerted markedly pro-proliferative and pro-migratory effects on endothelial cells compared to VCAM-1^−^CV-MSCs. Finally, transplantation of VCAM-1^+^CV-MSCs into the ischemic hind limb of BALB/c nude mice resulted in a significantly functional improvement in comparison with VCAM-1^−^CV-MSCs transplantation.

**Conclusions:**

VCAM-1^+^CV-MSCs possessed a favorable angiogenic paracrine activity and displayed therapeutic efficacy on hindlimb ischemia. Our results suggested that VCAM-1^+^CV-MSCs may represent an important subpopulation of MSC for efficient therapeutic angiogenesis.

**Electronic supplementary material:**

The online version of this article (doi:10.1186/s13287-016-0297-0) contains supplementary material, which is available to authorized users.

## Introduction

Peripheral arterial disease (PAD), characterized by the critical limb ischemia (CLI) with high morbidity and mortality risks, is gradually becoming an urgent life-threatening disease in our aging society. To date, the main treatments for PAD are bypass grafting and endarterectomy. However, surgery has not always been allowed [1]. Numerous studies have demonstrated that mesenchymal stem cells (MSCs) derived from different tissue sources exert therapeutic efficacy on ischemia [2–5]. Varieties of reports have highlighted the therapeutic angiogenesis of MSCs by focusing on differentiation and paracrine mechanisms [6]. Several angiogenic cytokines and enzymes secreted by MSCs, including vascular endothelial cell growth factor (VEGF)-A [7], hepatocyte growth factor (HGF) [8], interleukin (IL)-8 [9], transforming growth factor beta (TGFβ) [10], matrix metalloproteinases (MMPs) [11], and so forth, have been widely reported to initiate angiogenesis. Based on their angiogenic properties, MSCs are attractive in various clinical trials [12]. However, MSCs have been known to be heterogeneous [13, 14] and it remains to be determined whether some MSC subpopulations exert superior angiogenic activities and are more suitable for therapeutic angiogenesis.

Vascular cell adhesion molecule 1 (VCAM-1), also known as CD106, is extensively expressed on endothelial cells [15], and is also constitutively expressed on some stromal cells, existing in a particular vascular niche [16]. VCAM-1 plays a critical role in early embryonic development since VCAM-1-deficient mice often die early or show multiple severe defects in placental development [17]. In addition, soluble VCAM-1 (sVCAM-1) has shown evidence of mediating angiogenesis in rat cornea [18] and the sVCAM-1/α4 integrin pathway plays an important role in inflammatory stimuli-induced angiogenesis [19]. Recent studies demonstrated that VCAM-1 overexpression was associated with tumor angiogenesis, such as gastric carcinoma [20], breast cancer [21], and renal cancer [22]. These studies suggest VCAM-1 may be involved in angiogenesis.

We have previously isolated a VCAM-1^+^ MSC subpopulation of placenta chorionic villi (CV) that displayed unique immunomodulation capacity. VCAM-1^+^CV-MSCs secreted not only inflammatory factors but also angiogenic cytokines [23]. The aim of this work was to assess the angiogenic potential of the VCAM-1^+^CV-MSC subpopulation, and to explore its therapeutic application in an animal model of vascular ischemic disease.

## Methods

### Cell isolation and culture

This study was approved by the Ethical Committee and the Institutional Review Board of the Chinese Academy of Medical Science and Peking Union Medical College, Tianjin, China. All volunteers provided informed consent. CV-MSCs were harvested and cultured as described previously [23]. The regular culture medium for CV-MSCs was DF12 medium (Gibco, Grand Island, NY, USA), 10 % fetal bovine serum (FBS), 10 ng/ml epidermal growth factor (EGF; Peprotech, Rocky Hill, NJ, USA), 2 mM glutamine (Sigma, St.Louis, MO, USA), 1 % nonessential amino acids (Gibco), and 100 U/ml penicillin–streptomycin (Invitrogen, Carlsbad, CA, USA). Human umbilical vein endothelial cells (HUVECs) were harvested by digesting umbilical vein with 0.25 % trypsin (Gibco) for 15 minutes at 37 °C. The HUVECs were then cultured in EGM2-MV (Lonza, Walkersville, MD, USA).

### Flow cytometry analysis

The phenotype of CV-MSCs was analyzed using the following antibodies: phycoerythrin (PE)-conjugated CD105, CD73, CD166, CD29, CD54, VCAM-1, CD14, CD144, and CD133; and fluorescein isothiocyanate (FITC)-conjugated CD90, CD45, HLA-ABC, HLA-DR, and CD31. PE or FITC isotype-matched antibodies served as controls. Cells were examined by LSRII flow cytometer (BD Bioscience, San Jose, CA, USA ). For cell sorting, CV-MSCs were stained with PE-anti VCAM-1 antibodies for 30 minutes on ice before cell sorting using the BD FACS Aria III cell sorter (BD Biosciences, San Jose, CA, USA). All of the antibodies were purchased from BD Pharmingen (San Diego, CA, USA), and the flow cytometry data were analyzed by FlowJo 7.6 software (San Carlos, CA, USA).

### RNA extraction, reverse transcription, and real-time PCR

Total RNA was extracted using the E.Z.N.A. Total RNA Kit I (OMEGA, Norcross, GA, USA), and cDNA synthesis was performed using the MLV RT kit (Invitrogen). All of the procedures followed the manufacturer’s instructions. Real-time PCR was performed on an Applied Bio system 7900 Real-Time PCR System (Foster City, CA, USA), using a SYBR Green-based real-time detection method. Primers used are shown in Additional file 1: Table S1. Each sample was performed in triplicate.

### Tubular network formation assay in vitro

Pairs of VCAM-1^+^CV-MSCs and VCAM-1^−^CV-MSCs were seeded at 2 × 10^4^ cells/well gently on a Matrigel-coated (BD Biosciences, Bedford, MA, USA) 96-well plate. Photographs were taken by Microscope (Olympus, Melville, NY, USA) 12 hours later. Tube numbers in each well were counted. Three pairs of VCAM-1^+^CV-MSCs and VCAM-1^−^CV-MSCs were used, and each sample was performed in triplicate.

### Matrigel plug angiogenesis assay in vivo

Six-week-old nude male mice were purchased from the Institute of Experimental Animal (Beijing, China). All of the animal experiments followed the Peking Union Medical College Animal Care and Use Committee guidelines. VCAM-1^+/−^CV-MSCs or nonseparated (NS) CV-MSCs (10^6^ cells) were suspended in 400 μl Matrigel and injected subcutaneously into the dorsal area of nude mice. Matrigel supplement with phosphate-buffered saline (PBS) served as the negative control. Each group contained three to six mice. Three weeks later, Matrigel implants were harvested, photographed, fixed, sliced, and stained with hematoxylin and eosin (H & E; Sigma). Vessel numbers were counted under the microscope. Frozen slices stained with alpha-smooth muscle actin (α-SMA; Invitrogen) and von Willebrand factor (vWF; Abcam, Cambridge, MA, USA) were employed to detect the neovascular structures in the Matrigel plug. Photographs were taken at × 20 and × 60 objectives by confocal microscopy (UltraView; Perkin-Elmer, Waltham, Massachusetts, USA).

### Conditioned medium preparation and proliferation assay

Pairs of 10^6^ VCAM-1^+/−^CV-MSCs were incubated in EBM2 medium (Lonza) for 48 hours. Then their conditioned mediums (CMs) were collected, centrifuged at 1800 rpm for 10 minutes to remove cell debris, filtered through 0.2 μm filters (Pall Corporation, Ann Arbor, MI, USA), and frozen at –80 °C. To determine the pro-proliferative effect, VCAM-1^+/−^CV-MSC^CM^ supplemented with 2 % FBS were used to culture HUVECs for 72 hours. EBM2 supplemented with 2 % FBS, and EGM2-MV (endothelial cells commercial culture medium; Lonza) served as the negative and positive control, respectively. The Cell Counting Kit 8 (Dojindo, Rockville, MD, USA) method was used to measure HUVEC proliferation at 24, 48, and 72 hours. ΔOD450 indicated the final data after subtracting the background. Each sample was performed in quadruplicate.

### Scratch wound healing assay

When endothelial cells reached confluence, a scratch wound was generated across each well using a pipette tip. After washing with PBS, pairs of CM supplemented with 2 % FBS, EGM2-MV, or EBM2 + 2 % FBS were used to culture endothelial cells for 18 hours. The cleared area of each well was photographed under × 40 magnification at 0 and 18 hours, and measured by ImageJ software (NIH, USA). The percentage of area repopulation was calculated by the following formula:

$$ \begin{array}{l}\%\ \mathrm{of}\ \mathrm{area}\ \mathrm{repopulation} = \left(1 - \mathrm{clear}\ \mathrm{area}\ \mathrm{of}\ 18\ \mathrm{hours}\right)\\ \quad\qquad\qquad\qquad\qquad\qquad / \left(\mathrm{clear}\ \mathrm{area}\ \mathrm{of}\ 0\ \mathrm{hours}\right) \end{array} $$

Three pairs of CM were used and each sample was performed in triplicate.

### Enzyme-linked immunosorbent assay

The VEGF concentration in CM of VCAM-1^+^CV-MSCs and VCAM-1^−^CV-MSCs was measured using an enzyme-linked immunosorbent assay (ELISA; Neobioscience Biotech, Shenzhen, China). Each sample was measured in triplicate.

### Human cytokine antibody array

The human cytokine antibody array (AAH-CYT-G1000) was performed following the manufacturer’s instructions (RayBiotech, Norcross, GA, USA) to detect 120 cytokine expressions in supernatants (SN) of VCAM-1^+/−^CV-MSCs. Cytokine signals above 200 were further studied, and the cytokine signal ratio in VCAM-1^+^CV-MSCs and VCAM-1^−^CV-MSCs was calculated. This was statistically significant if the cytokine signal ratio was >1.3 or <0.75. Two pairs of VCAM-1^+^CV-MSCs and VCAM-1^−^CV-MSCs were used. Each sample was performed in duplicate. The targeted names of all cytokines involved are presented in Additional file 1: Table S2.

### Transplantation of VCAM-1^+/−^CV-MSCs in the hind limb ischemia model

Nude mice (male, 7–8 weeks old, 18–22 g) were intraperitoneally anesthetized with 100 mg/kg sodium pentobarbital (Sigma). Unilateral femoral artery ligation and excision were performed as described previously [24]. Nude mice were randomly divided into three groups (PBS, VCAM-1^+^CV-MSCs, and VCAM-1^−^CV-MSCs groups) after arterial ligations, and then 100 μl of a 10^6^ cell suspension or PBS was intramuscularly injected into ischemic hind limbs within 6 hours post surgery. Blood perfusion in ischemia and nonischemia limbs was measured by the PeriCam PSI System (PERIMED AB Company, Järfälla, stockholm, Sweden) on day 0, day 7, and day 20. Ischemia damage and functional assessment of ischemic hind limbs in each treatment group were assessed on day 20 according to the semiquantitative scores that had been described previously [24].

### Angiography

On day 20, after blood perfusion detection, mice were sacrificed for angiography to evaluate the vessel density in ischemic limbs. Angiographic images of hind limbs in three treatments were acquired by the Kodak In-Vivo FX ProImaging System (Kodak, New Haven, Connecticut, USA), and the angiography score was employed [24] to quantitatively analyze the collateral vessel formation at the ischemia site.

### Histological analysis

On day 20, after angiography, the ischemia adductor muscle of nude mice in each group was collected, fixed in 10 % formaldehyde (Sigma) overnight, and embedded in paraffin. To detect capillary densities in ischemic sites, H & E staining was performed and images were taken under × 200 magnification. Vessels containing barium sulfate or erythrocytes were counted, and the vessel density in each group was calculated and compared.

### Statistical analysis

Statistical analysis was performed using Graph Pad Prism 6.0 (Graph Pad Software, Inc., San Diego, CA, USA). All data are presented as mean ± standard error of the mean. The Mann–Whitney test and one-way analysis of variance (ANOVA) were performed to determine the significance. Fisher’s exact test (Freeman–Halton) was employed to assess the outcome of transplantation via a 3 × 3 contingency table. The difference was considered to be significant if *p* <0.05.

## Results

### Characteristics of CV-MSCs

**Fig. 1:**
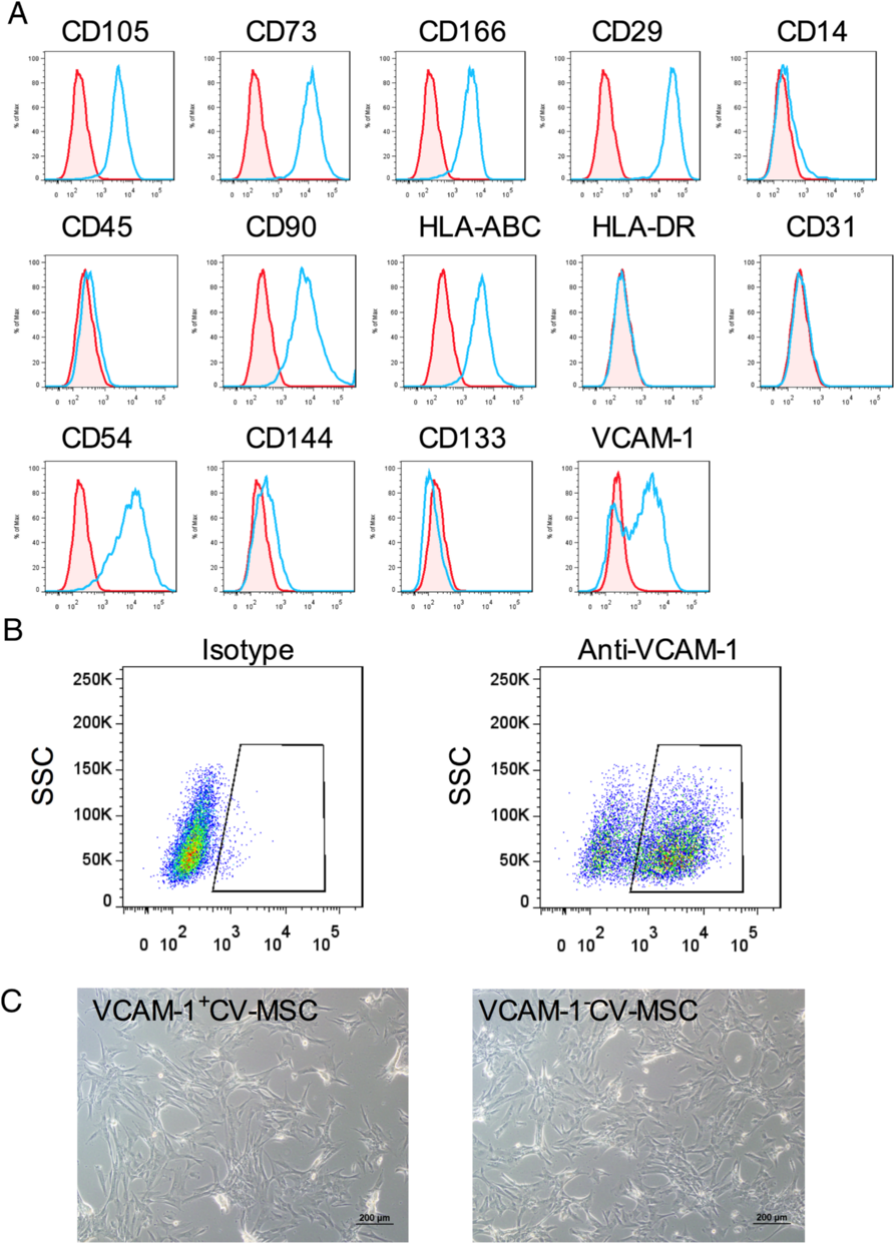
Phenotype of CV-MSCs and flow cell sorting. **a** Surface markers of CV-MSCs were evaluated by FACS analysis. CV-MSCs positively expressed CD105, CD73, CD166, CD29, CD90, HLA-ABC, CD54, and VCAM-1, and hardly expressed CD14, CD45, CD31, CD144, CD133 and HLA-DR. A representative sample is shown. **b** VCAM-1^+^CV-MSCs and VACM-1^−^CV-MSCs were separated by the BD Aria III cell sorting system. **c** Morphology of VCAM-1^+^CV-MSCs and VCAM-1^−^CV-MSCs (scale bar = 200 μm). *CV* chorionic villi, *MSC* mesenchymal stem cell, *SSC* side scatter, *VCAM-1* vascular cell adhesion molecule 1

CV-MSCs expressed high levels of CD105 (98.21 % ± 1.28 %), CD73 (99.22 % ± 0.05 %), CD166 (71.72 % ± 13.23 %), CD29 (99.69 % ± 0.14 %), CD90 (97.94 % ± 1.91 %), HLA-ABC (94.32 % ± 2.09 %), CD54 (80.87 % ± 8.25 %), and VCAM-1 (62.9 % ± 5.36 %), but hardly expressed endothelial cells markers (CD144, CD133, and CD31), the hematopoietic cell markers (CD14 and CD45), and immunogenic marker HLA-DR. FACS analysis of a representative sample is shown in Fig. 1a. Phenotypes of CV-MSCs derived from three distinct donors are presented in Additional file 1: Table S3. Cell sorting was carried out to separate the VCAM-1^+^CV-MSCs and VCAM-1^−^CV-MSCs (Fig. 1b), and the purity of cell sorting was greater than 90 %. VCAM-1^+^CV-MSCs and VCAM-1^−^CV-MSCs cultured in a flask showed typical spindle fibroblast-like shapes; no morphological difference was observed. Photographs of VCAM-1^+^CV-MSCs and VCAM-1^−^CV-MSCs are presented in Fig. 1c (scale bar = 200 μm).

### Angiogenic genes were highly expressed in VCAM-1^+^CV-MSCs

**Fig. 2:**
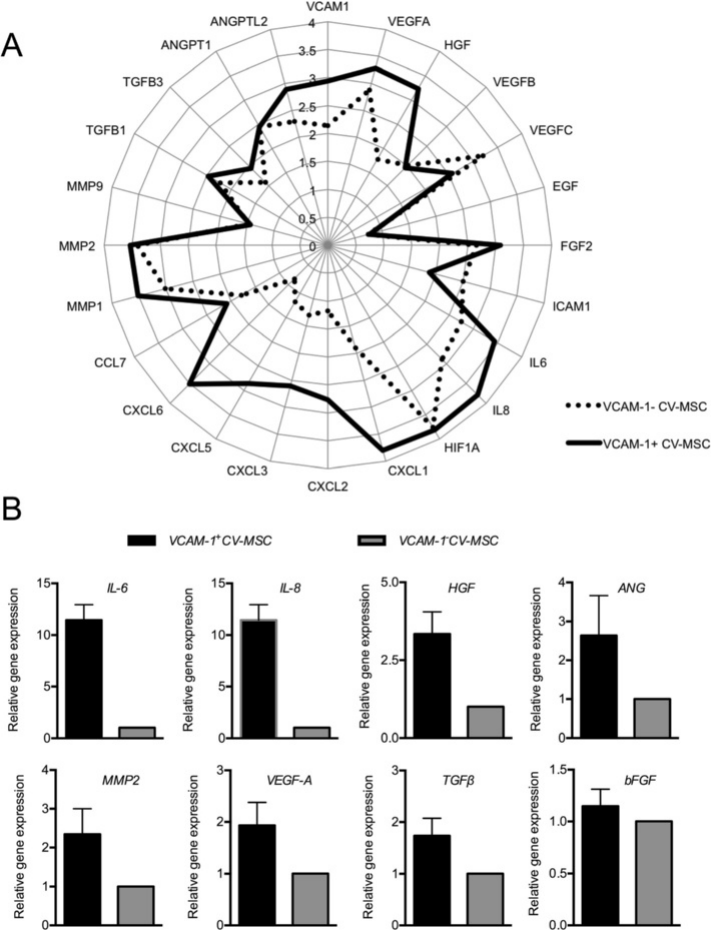
Angiogenic genes were upregulated in VCAM-1^+^CV-MSCs. **a** Gene expression profile of VCAM-1^+^CV-MSCs and VCAM-1^−^CV-MSCs determined using Affymetrix oligoarray, with the angiogenic genes valued and expressed in log_10_. **b** Several raised angiogenic genes in VCAM-1^+^CV-MSCs were confirmed by real-time PCR, including IL-6, IL-8 [23], HGF, ANG, MMP2, VEGF-A, TGFβ, and bFGF (*n* = 3–5). *ANG* angiogenin, *ANGPT2* angiopoietin-2, *ANGPTL2* angiopoietin-like protein 2, *BFGF* basic fibroblast growth factor, *CCL* Chemokine (C-C motif) ligand, *CV* chorionic villi, *CXCL* chemokine (C-X-C motif) ligand, *EGF* epidermal growth factor, *HGF* hepatocyte growth factor, *HIF* hypoxia-induced factor, *IL* interleukin, *MMP* matrix metalloproteinase, *MSC* mesenchymal stem cell, *TGF* transforming growth factor, *VCAM-1* vascular cell adhesion molecule 1, *VEGF* vascular endothelial cell growth factor

Our previous gene profile result indicated that VCAM-1^+^CV-MSCs expressed higher levels of angiogenic cytokines than VCAM-1^−^CV-MSCs, such as IL-6 (2.44-fold) and IL-8 (11.10-fold) [23]. Apart from that, the CXC chemokine family (chemokine (C-X-C motif) ligand (CXCL)1–CXCL3, CXCL5, and CXCL6 and chemokine (C-C motif) ligand (CCL7)), MMPs (including MMP1 and MMP2), several growth factors (VEGFA, HGF, basic fibroblast growth factor (bFGF), TGFβ1, and TGFβ3), hypoxia-induced factor (HIF1A), and angiopoietin-like protein 2 (ANGPTL2) were also highly expressed in VCAM-1^+^CV-MSCs. Meanwhile, the expressions of lymph-angiogenesis related VEGF-C and intercellular cell adhesion molecule-1 (ICAM-1) were lower in VCAM-1^+^CV-MSCs (Fig. 2a). Several critical angiogenic genes were further confirmed by real-time PCR. Results showed that HGF, angiogenin (ANG), MMP2, VEGFA, TGFβ, and bFGF expressed in VCAM-1^+^CV-MSCs were upregulated to varying degrees, with a 3.34-fold, 2.64-fold, 2.34-fold, 1.93-fold, 1.74-fold, and 1.14-fold increase compared with VCAM-1^−^CV-MSCs, respectively (*n* = 3–5; Fig. 2b).

### VCAM-1^+^CV-MSCs displayed angiogenic potential on Matrigel assay in vitro and in vivo

**Fig. 3:**
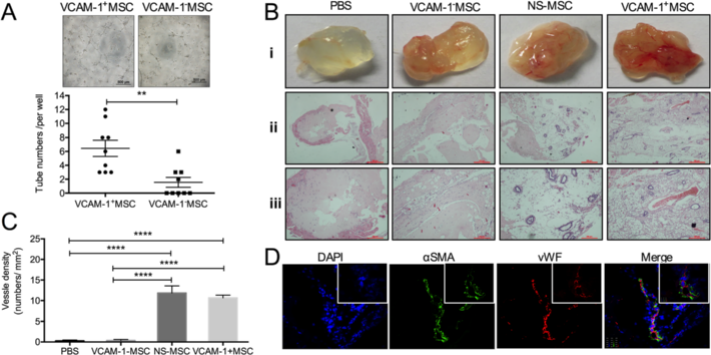
VCAM-1^+^CV-MSCs revealed vasculoangiogenic potential by angiogenesis analysis with Matrigel in vitro and vivo. **a** VCAM-1^+^CV-MSCs spontaneously formed much more intact tube-structures on Matrigel than VCAM-1^−^CV-MSCs (*n* = 3, ** *p* <0.01), indicating that VCAM-1^+^CV-MSCs possessed vasculogenic potential. Representative images are shown (scale bar = 500 μm). Each sample was performed in triplicate. **b** Macroscopic and microscopic view of Matrigel plugs. The Matrigel plug was harvested 21 days later; macroscopic vessels were seen in the Matrigel plug in the VCAM-1^+^CV-MSCs and NS CV-MSCs groups **i**. H & E staining was performed to reveal the vessel density in Matrigel plug (scale bar: **ii** = 500 μm; **iii** = 200 μm). **c** Vessel densities in the VCAM-1^+^CV-MSCs and NS CV-MSCs groups were much greater than in the PBS and VCAM-1^−^CV-MSCs groups (*n* = 3, **** *p* <0.0001). **d** New outgrowth in Matrigel plug was immunostained with vWF and α-SMA antibodies to indicate the endothelial cells and smooth muscle cells, respectively. Photographs were taken under × 20 (*bottom*) and × 60 (*upper*) magnifications. *α-SMA* alpha smooth muscle actin, *CV* chorionic villi, *DAPI* 4',6-diamidino-2-phenylindole, *MSC* mesenchymal stem cell, *NS* nonseparated, *PBS* phosphate-buffered saline, *VCAM-1* vascular cell adhesion molecule 1, *vWF* von Willebrand factor

To determine the angiogenic potential of VCAM-1^+^CV-MSCs and VCAM-1^−^CV-MSCs, a tubular network assay was performed in vitro. To our surprise, without exogenous VEGF, VCAM-1^+^CV-MSCs spontaneously formed about 4.14-fold intact tubular structures on Matrigel compared with VCAM-1^−^CV-MSCs (*n* = 3, *p* <0.01; Fig. 3a). Matrigel plug angiogenesis assays in vivo [25] were then performed to explore the angiogenic differences. Interestingly, plenty of macroscopic blood vessels were observed in the Matrigel plugs of the VCAM-1^+^CV-MSCs and NS CV-MSCs groups rather than the VCAM-1^−^CV-MSCs and PBS groups (Fig. 3b–i). H & E staining revealed that the new outgrowth contained erythrocytes and the smooth muscle layer (Fig. 3b ii, iii). Moreover, vessel densities in the VCAM-1^+^CV-MSCs and NS CV-MSCs groups were significantly higher than in the VCAM-1^−^CV-MSCs and PBS groups (10.66 ± 0.67 and 11.84 ± 1.23 per mm^2^ vs. 0.36 ± 0.24 and 0.27 ± 0.19 per mm^2,^*n* = 3, *p* <0.0001; Fig. 3c). However, the vessel density in the VCAM-1^+^CV-MSCs and NS CV-MSCs groups was similar (*p* >0.05). Besides that, a larger vessel lumen was observed in the VCAM-1^+^CV-MSCs group rather than in the NS CV-MSCs group, which could be related to a higher VCAM-1^+^CV-MSC proportion in the transplanted cells. Moreover, immunostaining of vWF and α-SMA revealed that the fresh blood vessels contained endothelial cells (labeled with anti-vWF antibodies) and smooth muscle cells (labeled with anti-α-SMA antibodies; Fig. 3d), indicating that the vessel structures were intact and mature.

### VCAM-1^+^CV-MSC^CM^ effectively promoted endothelial cell proliferation and migration

**Fig. 4:**
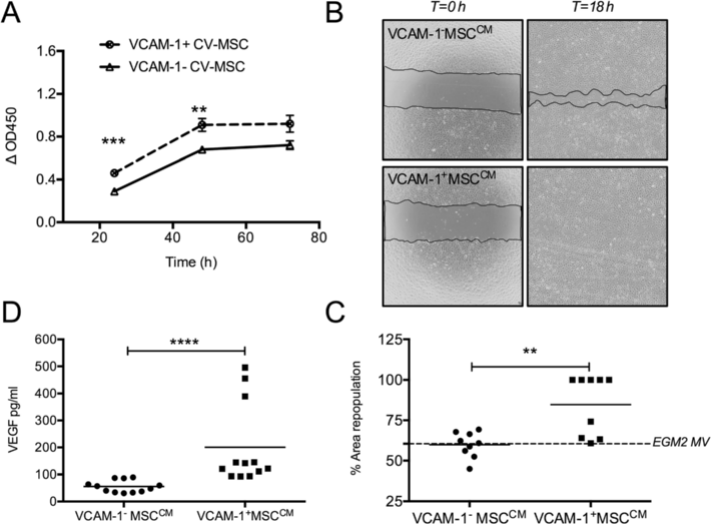
VCAM-1^+^CV-MSC^CM^ exerted angiogenic paracrine effects on endothelial cells. **a** Endothelial cell proliferation assay was used to study the pro-proliferative activity of VCAM-1^+^CV-MSCs and VCAM-1^−^CV-MSCs. By comparison with VCAM-1^−^CV-MSC^CM^, VCAM-1^+^CV-MSC^CM^ significantly promoted endothelial cell proliferation during 48 hours, but this effect was not significant at 72 hours (*n* = 3, **p <0.01, ***p <0.001). Each sample was done in quadruplicate. **b** Wound healing assay was performed to study the pro-migratory effect of VCAM-1^+/−^CV-MSCs. Representative photographs were shown at 0 and 18 hours under × 40 magnification. **c** Result of area repopulation (%) indicating that VCAM-1^+^CV-MSC^CM^ efficiently accelerated the endothelial cell wound healing process compared with VCAM-1^−^CV-MSC^CM^ (*n* = 3, **p <0.01). Each sample was performed in triplicate. **d** VEGF concentration in VCAM-1^+^CV-MSC^CM^ and VCAM-1^−^CV-MSC^CM^ measured by ELISA (*n* = 4, ****p <0.0001). Each sample was tested in triplicate. *CM* conditioned medium, *CV* chorionic villi, *MSC* mesenchymal stem cell, *VCAM-1* vascular cell adhesion molecule 1, *VEGF* vascular endothelial cell growth factor

To explore the paracrine activities of VCAM-1^+^CV-MSCs and VCAM-1^−^CV-MSCs, we collected their CMs and performed endothelial cell proliferation and scratch wound healing assay. Our data revealed that compared with the VCAM-1^−^CV-MSC^CM^, VCAM-1^+^CV-MSC^CM^ significantly promoted endothelial cell proliferation during 48 hours (*n* = 3, *p* <0.01), with the most significant point at 24 hours (*n* = 3, *p* <0.001). But this pro-proliferative effect was not significant after 72 hours (*n* = 3, *p* >0.05; Fig. 4a). The reason for this might be the exhaustion of angiogenic cytokines. In addition, scratch assay that mimicked the wound healing process in vitro was used to evaluate the pro-migratory effects. After incubation for 18 hours, we surprisingly found that endothelial cells cultured in VCAM-1^+^CV-MSC^CM^ reached confluence again. Representative photographs were taken under × 40 magnification and the percentage of area repopulation was calculated by Image J software (NIH, USA) (Fig. 4b). VCAM-1^+^CV-MSC^CM^ significantly increased the cleared area recovery compared with VCAM-1^−^CV-MSC^CM^ (80.58 ± 6.88 vs. 56.36 ± 4.23, *n* = 3, *p* <0.01; Fig. 4c), indicating that VCAM-1^+^CV-MSC^CM^ was richer in pro-migratory cytokines than VCAM-1^−^CV-MSC^CM^. To figure out the paracrine mechanism of VCAM-1^+^CV-MSCs, we performed VEGF and sVCAM-1 ELISAs. Results showed that the VEGF concentration in VCAM-1^+^CV-MSC^CM^ was 200 pg/ml, 3.6-fold higher than VCAM-1^−^CV-MSC^CM^ (*n* = 4, *p* <0.0001; Fig. 4d), while the sVCAM-1 concentration was <20 pg/ml (Additional file 1: Figure. S1). The fact that VEGF can induce endothelial cell proliferation and migration [7] may partially explain the pro-proliferative and pro-migratory differences between VCAM-1^+^CV-MSC^CM^ and VCAM-1^−^CV-MSC^CM^ on endothelial cells.

### Cytokine antibody array revealed the angiogenic secretome of VCAM-1^+^CV-MSCs

**Fig. 5:**
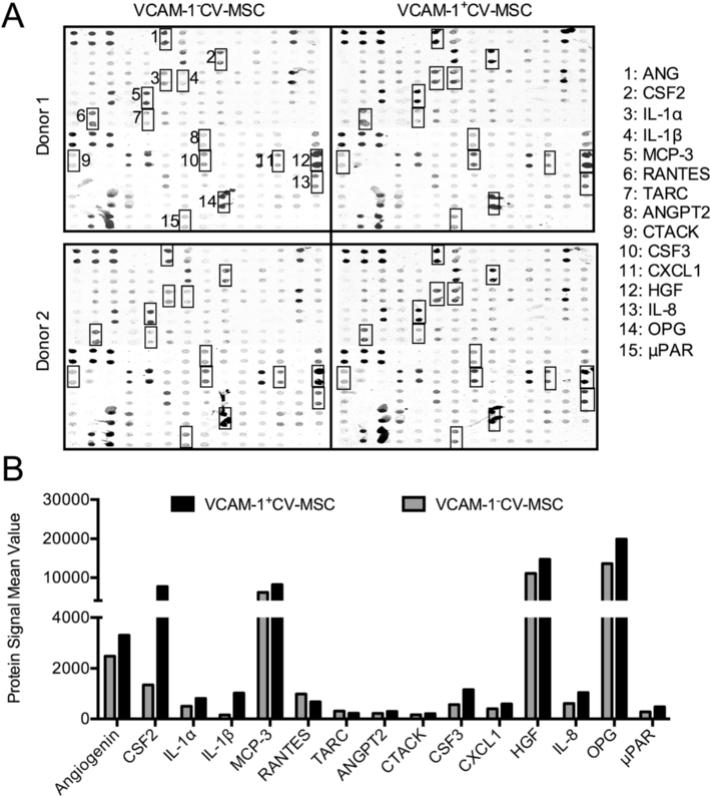
Human cytokine antibody array displayed the angiogenic secretome of VCAM-1^+^CV-MSCs. **a** Expression of 120 cytokines in SN of VCAM-1^+^CV-MSCs and VCAM-1^−^CV-MSCs was determined by human cytokine antibody array (AAH-CYT-G1000). VCAM-1^+^CV-MSC and VCAM-1^−^CV-MSC SN derived from two healthy donors were used. Each sample was performed in duplicate. The differential angiogenic cytokines between VCAM-1^+^CV-MSCs and VCAM-1^–^CV-MSCs were similar in two healthy donors. **b** Cytokine signal >200 was analyzed, and ratio of cytokine signal in VCAM-1^+^CV-MSCs to VCAM-1^−^CV-MSCs was calculated. This was statistically significant if the cytokine signal ratio was >1.3 or <0.75. Data revealed that VCAM-1^+^CV-MSCs secreted more abundant angiogenic cytokines than VCAM-1^–^CV-MSCs, including HGF, IL-8, ANG, ANGPT2, μPAR, CXCL1, IL-1β, IL-1α, CSF2, CSF3, MCP-3, CTACK, and OPG. *CV* chorionic villi, *MSC* mesenchymal stem cell, *VCAM-1* vascular cell adhesion molecule 1. See Abbreviations for cytokine definitions

To systematically study the secretome of VCAM-1^+^CV-MSCs and VCAM-1^−^CV-MSCs, we performed the human cytokine antibody array (AAH-CYT-G1000). Our data revealed that: the differential cytokines from the two donors were similar between VCAM-1^+^CV-MSCs and VCAM-1^−^CV-MSCs (Fig. 5a); the VCAM-1^+^CV-MSC secretome contained a significantly higher level of angiogenic cytokines (Fig. 5b, Additional file 1: Table S4), including IL-1β (6.57-fold), hematopoietic colony-stimulating factor 2 (CSF2)/granulocyte–macrophage colony-stimulating factor (GM-CSF, 5.73-fold), CSF3/granulocyte colony-stimulating factor (G-CSF; 2.03-fold), IL-8 (1.70-fold), CXCL1 (Growth regulated oncogene-α (GRO-α), 1.48-fold), osteoprotegerin (OPG, 1.46-fold), urokinase-type plasminogen activator receptor (μPAR, 1.69-fold), IL-1α (1.6-fold), angiopoietin-2 (ANGPT2, 1.35-fold), HGF (1.33-fold), ANG (1.33-fold), monocyte chemotactic protein-3 (MCP-3/CCL-7, 1.31-fold), and cutaneous T-cell attracting chemokine (CTACK/CCL27, 1.30-fold), some of those differential cytokines consistent with our gene profile results; and the secretion of RANTES (0.68-fold) and TARC (0.74-fold) was lower in VCAM-1^+^CV-MSCs than in VCAM-1^–^CV-MSCs (Fig. 5b). Because of a signal value less than 200, VEGF was not the principal cytokine secreted by CV-MSCs in normal conditions.

### VCAM-1^+^CV-MSCs exerted therapeutic efficacy on hind limb ischemia

**Fig. 6:**
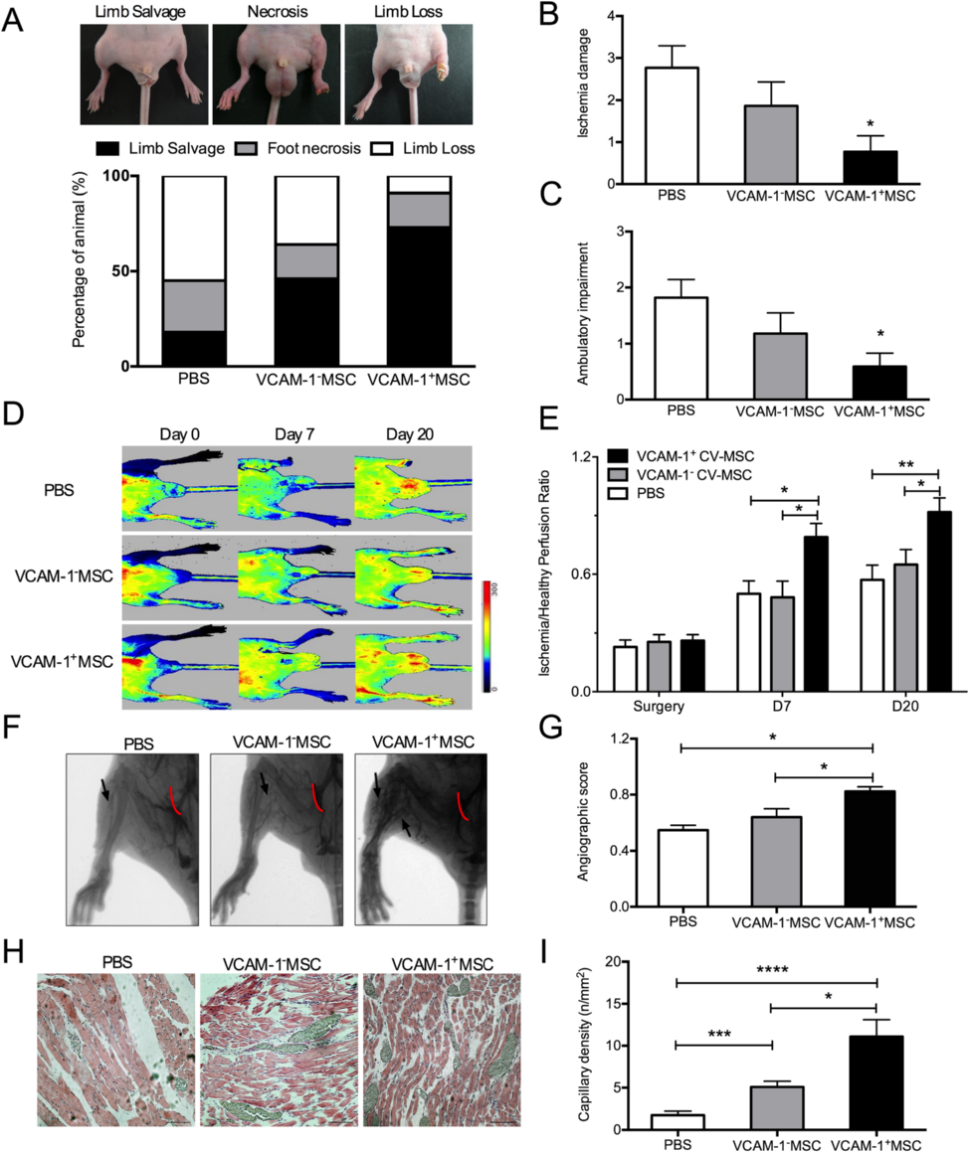
Transplantation of VCAM-1^+^CV-MSCs significantly enhanced the blood perfusion and the generation of collateral vessels in the ischemic sites. **a** VCAM-1^+/−^CV-MSCs or PBS were injected into the ischemic site of nude mice. Percentage distributions of limb salvage, foot necrosis, and limb loss in the three groups are shown and analyzed by the Fisher’s exact test (*n* = 11, *p* = 0.102). Ischemia damage and physiological function of ischemic limbs were semiquantified by ischemia scores **b** and ambulatory impairment scores **c** (*n* = 11, *p <0.05). **d** Blood perfusion in ischemic/healthy limb was detected by the Kodak In-Vivo FX Pro Imaging System on days (*D*) 0, 7, and 20. Different colors indicate different blood perfusion. Blood flow increased from *dark blue* to *red*. **e** Blood perfusion ratio in ischemic to healthy limbs was used to quantitatively analyze the blood flow restoration in ischemic limbs (*n* = 11, *p <0.05, **p <0.01). **f** Angiography was performed to assess the collateral vessel generation in the ischemic site. *Red curves* indicated the site of the femoral arteries incision; *black arrows* showed the collateral vessels in the ischemic hind limb. **g** Angiography score indicated that VCAM-1^+^CV-MSCs were superior to VCAM-1^−^CV-MSCs in augmenting collateral vessels (*n* = 3–5, *p <0.05). **h** H & E staining was performed to study the vessel density in ischemia limbs. Pictures showed that the blood vessels were full of barium sulfate (*silver*, scale bar = 100 μm). **i** Vessel density in VCAM-1^+^CV-MSC or VCAM-1^−^CV-MSCs group was significantly greater than the PBS group (*n* = 7, ***p <0.001, ****p <0.0001). Furthermore, VCAM-1^+^CV-MSC transplantation apparently promoted the vessel generation compared with the VCAM-1^−^CV-MSCs group (**p <0.05). *CV* chorionic villi, *MSC* mesenchymal stem cell, *PBS* phosphate-buffered saline, *VCAM-1* vascular cell adhesion molecule 1 (Color figure online)

To investigate the therapeutic neovascularization of VCAM-1^+^CV-MSCs, we constructed a vascular ischemia animal model and intramuscularly injected 10^6^ VCAM-1^+/−^CV-MSCs into the ischemic limbs within 6 hours post surgery. PBS served as a negative control. To estimate the therapeutic effect, we classified mice into three outcomes: limb salvage, foot necrosis, and limb loss. Different percentage distributions of outcomes among three groups were calculated and Fisher’s exact test (Freeman–Halton) was used to analyze this result (*p* = 0.10, *n* = 11; Fig. 6a). From the data, mice in the PBS group suffered the maximal amputation rate (54.5 %) and foot necrosis rate (27 %). The amputation rate in the VCAM-1^−^CV-MSCs group was much higher than in the VCAM-1^+^CV-MSCs group (36.4 % vs. 9 %), while the foot necrosis rate in both of them was 18.2 %. Semiquantitative scores of ischemia damage and ambulatory impairment were used to assess ischemic states and physiological function of ischemic limbs. Results indicated that VCAM-1^+^CV-MSCs significantly alleviated the ischemia damage and ambulatory impairment (0.77 ± 0.37 and 0.59 ± 0.24), much better than the PBS group (2.77 ± 0.52 and 1.82 ± 0.33, *n* = 11, *p* <0.05), while VCAM-1^−^CV-MSCs showed a slight improvement compared with PBS treatment (1.86 ± 0.57 and 1.18 ± 0.36, *n* = 11, *p* >0.05; Fig. 6b, c).

In addition, blood perfusion detected by the PeriCam PSI System was utilized to evaluate ischemia restoration on days 0, 7 and 20 post surgery (Fig. 6d). The blood perfusion ratio in ischemic and healthy limbs was calculated and compared by ANOVA Bonferroni’s multiple test. Data showed a significant blood flow increased on day 7 and day 20 post surgery in the VCAM-1^+^CV-MSCs group (0.79 ± 0.07 and 0.92 ± 0.07), much higher than the VCAM-1^−^CV-MSCs group (0.48 ± 0.08 and 0.65 ± 0.08, *n* = 11, *p <0.05) and the PBS group (0.50 ± 0.07 and 0.57 ± 0.07, *n* = 11, *p <0.05, **p <0.01; Fig. 6e). By comparison, VCAM-1^−^CV-MSCs did not show similar therapeutic effects as VCAM-1^+^CV-MSCs (*p* >0.05; Fig. 6e). To study collateral vessel development at the ischemic site, mice underwent angiography and in vivo images on day 20 post surgery. Representative photographs are shown in Fig. 6f. The angiography score that described vessel density was used to analyze the neovascularization. Results demonstrated that VCAM-1^+^CV-MSCs significantly augmented the generation of collateral vessels at the ischemia site (*n* = 3–5, *p <0.05; Fig. 6g), whose angiography score was 1.52-fold and 1.28-fold higher than the PBS and VCAM-1^−^CV-MSCs groups, respectively. H & E staining further confirmed that the vessel density in the VCAM-1^+^CV-MSCs group was 6.3-fold and 2.17-fold more than the PBS and VCAM-1^−^CV-MSCs groups (*n* = 7, *p <0.05, ***p <0.001, ****p <0.0001; Fig. 6h, i).

## Discussion

Previous studies have reported that MSCs displayed remarkable therapeutic properties on vascular ischemic diseases such as myocardial infarction, stroke, and perivascular ischemic diseases [12]. However, the mechanisms of therapeutic angiogenesis induced by MSCs have not yet been well defined. Several investigators have proposed that paracrine factors secreted from MSCs, including a core of angiogenic cytokines (i.e., VEGF, HGF, IL-8, TGFβ), exosomes [26], and microvesicles [27], might be the major contributors [28]. Gnecchi et al. [29] reported that injection with the CM of Akt-modified MSCs abundant with VEGF, bFGF, HGF, and TB4 significantly improved cardiac performance after induced myocardial infarction. Recent studies using cell labeling [30] and single cell technology [31] also supported the major status of paracrine action in MSC-mediated angiogenesis.

In this study, we have firstly demonstrated that the VCAM-1^+^CV-MSC subpopulation displayed a potent angiogenic property and exerted enhanced therapeutic efficacy on regeneration after ischemia in comparison with the VCAM-1^−^CV-MSC subpopulation.

We then wanted to know why VCAM-1^+^CV-MSCs possessed superior pro-angiogenic activities than VCAM-1^−^CV-MSCs. We were interested to note a superior angiogenic secretome from VCAM-1^+^CV-MSCs, including HGF, IL-8, ANG, ANGPT2, CXCL1/GRO-α, μPAR, IL-1β, IL-1α, CSF2/GM-CSF, CSF3/G-CSF, MCP-3, CTACK/CCL27, and OPG. Previous studies have shown that HGF potently stimulated endothelial cell motility and growth [32]. IL-8 promoted angiogenesis via directly enhancing endothelial cell proliferation, survival, and MMP production [33]. ANG potently induced new blood vessel formation [34]. ANGPT-2 potentiated the effects of other angiogenic cytokines in vivo and initiated neovascularization [35]. CXCL1 enhanced microvascular endothelial cell migration and tube formation [36]. μPAR induced endothelial cell invasion and proliferation in the initial period of angiogenesis [37]. IL-1β [38], IL-1α [39], GM-CSF [40], and G-CSF [41] were reported to initiate angiogenesis by stimulating VEGF production or activating the angiogenesis-related pathway. MCP-3 stimulated the migration of circulating angiogenic cells and angiogenesis partially via the chemokine (C-X-C motif) receptor 1 (CCR1) [42]. CTACK/CCL27 was reported to accumulate the CD34^+^ bone marrow cells (expressing CCR10) to participate in skin wound healing and repair [43]. OPG was a positive regulator of microvessel formation in vivo and could activate endothelial colony-forming cells [44]. In addition, we have performed the endothelial cell differentiation assay in vitro and have not found significant differences between VCAM-1^+^CV-MSCs and VCAM-1^−^CV-MSCs (seen by immunostaining of vWF) under a confocal microscope (Additional file 1: Figure. S2). Based on these studies, we believed that paracrine action rather than differentiation was the principal mechanism of the therapeutic angiogenesis induced by MSCs. Besides, the superior angiogenic effect of VCAM-1^+^CV-MSCs could be a result of a synergic effect of multiple angiogenic factors secreted by cells.

To date, the identification of MSC still relies on the minimal criteria specified in 2006 (plastic adhesion, expressing a set of membrane antigens and tridifferentiation capacities) [45]. Besides these properties, the trait of MSCs varies among different origins and individuals; that is, the paracrine actions [46], and immunomodulatory [23] and hematopoietic support capacities [47]. In addition, MSCs isolated from the same tissue also comprised a heterogeneous population. A variety of markers (i.e., Stro-1, SSEA-4, CD271, CD146) have hence been adopted to investigate the potential of particular MSC subpopulations [14]. Psaltis et al. [48] reported that stro-1^+^ bone marrow-derived MSCs possessed unique cardiovascular paracrine activities. Interestingly, Gronthos et al. [49] employed VCAM-1 as a coexpressed maker to enrich stro-1^+^ MSCs. Our data agree with Psaltis et al.’s study, which verified the consistent angiogenic potentials of VCAM-1^+^ MSCs. Most recently, Wang et al. [50] reported that MSCs pretreated with IL-1β and tumor necrosis factor alpha could enhance the therapeutic efficacy on cardiovascular ischemia via upregulating VCAM-1 expression. Consistently, our study demonstrated the presence of a natural VCAM-1^+^ MSC subpopulation in vivo in placenta CV that exerted excellent paracrine action. Additionally, it has been shown that placenta CV and bone marrow abundant with capillaries contained many more VCAM-1^+^ MSCs (68 % and 13 %) than adipose tissue and umbilical cord (0.24 % and 4 %) [23], suggesting that VCAM-1^+^ MSCs might play important roles in the physiological vasculogenesis and angiogenesis.

## Conclusion

Our comparative studies at multiple levels on the angiogenic properties of VCAM-1^+^CV-MSCs and VCAM-1^–^CV-MSCs showed that VCAM-1 could be used as a surface marker to select a MSC subpopulation with superior pro-angiogenic activity. Moreover, the exciting therapeutic efficacy of VCAM-1^+^CV-MSCs on ischemic nude mice not only provided a novel strategy for cell-based therapy of ischemic diseases, but also a hint for banking appropriate MSCs for clinical usage.

## Additional file

Additional file 1: is Table S1 presenting primers for real-time reverse transcription PCR, Table S2 presenting the description of the human cytokine antibody array (AAH-CYT-G1000), Table S3 presenting the phenotype of three donor-derived CV-MSCs, Table S4 presenting differential angiogenesis cytokines of VCAM-1^–^CV-MSCs and VCAM-1^+^CV-MSCs, Fig. S1 showing sVCAM-1 concentration in 48-hour CM of VCAM-1^+^CV-MSCs and VCAM-1^–^CV-MSCs measured by ELISA, and Fig. S2 showing the endothelial-like cells derived from VCAM-1^+^CV-MSCs and VCAM-1^–^CV-MSCs harvested after in vitro endothelial induction and immunostained by anti-vWF antibodies to evaluate their endothelial differentiation capacities. (PDF 524 kb)

## Abbreviations

α-SMA: Alpha-smooth muscle actin
ANG: Angiogenin
ANGPT2: Angiopoietin-2
ANGPTL2: Angiopoietin-like protein 2
ANOVA: Analysis of variance
bFGF: Basic fibroblast growth factor
CCL: Chemokine (C-C motif) ligand
CCR: Chemokine (C-C motif) receptor
CLI: Critical limb ischemia
CM: Conditioned medium
CSF: Colony-stimulating factor
CTACK: Cutaneous T-cell attracting chemokine
CV: Chorionic villi
CXCL: Chemokine (C-X-C motif) ligand
EGF: Epidermal growth factor
ELISA: Enzyme-linked immunosorbent assay
FACS: Fluorescence-activated cell sorting
FBS: Fetal bovine serum
FITC: Fluorescein isothiocyanate
G-CSF: Granulocyte-colony stimulating factor
GM-CSF: Granulocyte–macrophage colony-stimulating factor
GRO-α: Growth regulated oncogene-α
H & E: Hematoxylin and eosin
HGF: Hepatocyte growth factor
HIF: Hypoxia-induced factor
HUVEC: Human umbilical vein endothelial cell
ICAM-1: Intercellular cell adhesion molecule-1
IL: Interleukin
MCP: Monocyte chemotactic protein
MMP: Matrix metalloproteinase
MSC: Mesenchymal stem cell
NS: Nonseparated
OPG: Osteoprotegerin
PAD: Peripheral arterial disease
PBS: Phosphate-buffered saline
PE: Phycoerythrin
RANTES: Regulated on activation, normal T cell expressed and secreted
SN: Supernatants
sVCAM-1: Soluble vascular cell adhesion molecule 1
TARC: Thymus and activation regulated chemokine
TGFβ: Transforming growth factor beta
μPAR: Urokinase-type plasminogen activator receptor
VCAM-1: Vascular cell adhesion molecule 1
VEGF: Vascular endothelial cell growth factor
vWF: Von Willebrand factor
