# Supplementary material for: VCAM-1+ placenta chorionic villi-derived mesenchymal stem cells display potent pro-angiogenic activity
Source: Stem Cell Res Ther. 2016 Apr 4;7:49. doi: 10.1186/s13287-016-0297-0 (PMC4820943; doi:10.1186/s13287-016-0297-0)

VCAM-1$^+$ placenta chorionic villi derived Mesenchymal stem cells display potent pro-angiogenic activity

Wenjing Du, Xue Li, Ying Chi, Fengxia Ma, Zongjin Li, Shaoguang Yang, Baoquan Song, Junjie Cui, Tao Ma, Juanjuan Li, Jianjian Tian, Zhouxin Yang, Xiaoming Feng, Fang Chen, Shihong Lu, Lu Liang, Zhi-bo Han and Zhong-chao Han

Supplemental Methods and Results

Soluble VCAM-1 ELISA assay

Soluble VCAM-1 (sVCAM-1) concentration in conditioned medium (CM) of VCAM-1$^+$ and VCAM-1$^-$CV-MSCs were measured by using enzyme-linked immunosorbent assay (Neobioscience Biotech). Each sample was performed in triplicates.

Endothelial-like cells differentiation assay *in vitro*

VCAM-1$^+$ and VCAM-1$^-$CV-MSCs were seeded on Matrigel (1:100 dilution, BD) pre-coated flasks and induced endothelial-differentiation in EGM2-MV (Lonza) supplemented with 50ng/ml VEGF (Prepotech) for two weeks. Induction medium was changed twice a week. Cells after induction were collected and assessed by immunostaining of vWF (1:100 dilution, Abcam). The immunostaining procedure as following: cells were fixed with 4% paraformaldehyde (PFA) for 15 minutes, and then permeabilized by 0.25% Triton solution for 15 minutes. After blocking the non-specific

sites with 0.2% bovine serum albumin (BSA) for 30 minutes, cells were immunostained with dilulated mouse anti-human vWF for 30 minutes; later cells were washed by PBS and incubated with goat anti-mouse antibodies (1:100 dilution; Invitrogen, Molecular Probes) for another 30minutes. Cells stained with the second antibodies were used as control. Then, Rhodamine phalloidin (1:300 dilution; Life Technologies) was used to stain cells for 30 minutes before DAPI staining. Photos were taken by using Confocal Microscope (Leica).

Supplemental Tables

Table S1: Primers for real time reverse transcription-polymerase chain reaction.

Primers	Sequence
Human GAPDH forward:	5’-CGGATTTGGTCGTATTGGGC -3’
Human GAPDH reverse:	5’-CTTCCCGTTCTCAGCCTTG -3’
Human VEGF-A forward	5’-AGGGCAGAATCATCACGAAGT-3’
Human VEGF-A reverse:	5’-AGGGTCTCGATTGGATGGCA-3’
HumanVCAM-1forward:	5’-GGGAAGATGGTCGTGATCCTT-3’
HumanVCAM-1 reverse:	5’-TCTGGGGTGGTCTCGATTTTA-3’
Human HGF forward:	5’-GCTATCGGGGTAAAGACCTACA-3’
Human HGF reverse:	5’-CGTAGCGTACCTCTGGATTGC-3’
Human bFGF forward:	5’-AGTGTGTGCTAACCGTTACCT-3’
Human bFGF reverse:	5’-ACTGCCCAGTTCGTTTCAGTG-3’
Human MMP2 forward:	5’-GATACCCCTTTGACGGTAAGGA-3’
Human MMP2 reverse:	5’-CCTTCTCCCAAGGTCCATAGC-3’
Human TGFβ forward:	5’-GGCCAGATCCTGTCCAAGC-3’
Human TGFβ reverse:	5’-GTGGGTTTCCACCATTAGCAC-3’
Human ANG forward:	5’-CAAGGCCATCTGTGAAAACAAG-3’
Human ANG reverse:	5’-CAGGGGGAACCTCCATGTAG-3’
Human IL-6 forward:	5’-CCACACAGACAGCCACTCAC-3’
Human IL-6 reverse:	5’-CCAGATTGGAAGCATCCATC-3’
Human IL-8 forward:	5’-TTGGCAGCCTTCCTGATTT-3’
Human IL-8 reverse:	5’-TCAAAAACTTCTCCACAACCC-3’

120 cytokines expression in supernatants of VCAM-1^{+} and VCAM-1^{-}CV-MSCs were detected. Two donors derived VCAM-1$^{+/-}$ CV-MSCs were used. Each sample was performed in duplicates.

Further information: http://www.raybiotech.com/human-cytokine-array-g1000-4.html

Table S2: The description of the Human cytokine antibody array (AAH-CYT-G1000)

A	B	C	D	E	F	G	H	I	J	K	L	M	N
POS1	POS2	POS3	NEG	NEG	ANG	BDNF	BLC	BMP-4	BMP-6	Ckβ8-1	CNTF	EGF	CCL11
POS1	POS2	POS3	NEG	NEG	ANG	BDNF	BLC	BMP-4	BMP-6	Ckβ8-1	CNTF	EGF	CCL11
CCL24	CCL26	FGF6	FGF7	Flt-3L	CX3CL1	GCP-2	GDNF	CSF2	I-309	IFN-γ	IGFBP1	IGFBP2	IGFBP4
CCL24	CCL26	FGF6	FGF7	Flt-3L	CX3CL1	GCP-2	GDNF	CSF2	I-309	IFN-γ	IGFBP1	IGFBP2	IGFBP4
IGF-1	IL-10	IL-13	IL-15	IL-16	IL-1α	IL-1β	IL-1ra	IL-2	IL-3	IL-4	IL-5	IL-6	IL-7
IGF-1	IL-10	IL-13	IL-15	IL-16	IL-1α	IL-1β	IL-1ra	IL-2	IL-3	IL-4	IL-5	IL-6	IL-7
Leptin	LIGHT	MCP1	MCP2	MCP3	MCP4	M-CSF	MDC	MG	MIP-1δ	MIP-3α	NAP-2	NT-3	PARC
Leptin	LIGHT	MCP1	MCP2	MCP3	MCP4	M-CSF	MDC	MG	MIP-1δ	MIP-3α	NAP-2	NT-3	PARC
PDGF-BB	RANTES	SCF	SDF-1	TARC	TGF-β1	TGF-β3	TNF-α	TNF-β	NEG	NEG	NEG	NEG	NEG
PDGF-BB	RANTES	SCF	SDF-1	TARC	TGF-β1	TGF-β3	TNF-α	TNF-β	NEG	NEG	NEG	NEG	NEG
POS1	POS2	POS3	NEG	NEG	Acrp30	AgRP	ANGPT2	AREG	Axl	bFGF	βNGF	BTC	CCL28
POS1	POS2	POS3	NEG	NEG	Acrp30	AgRP	ANGPT2	AREG	Axl	bFGF	βNGF	BTC	CCL28
CTACK	Dtk	EGFR	ENA-78	Fas	FGF4	FGF9	CSF3	GITRL	GITR	GRO	GRO-α	HCC-4	HGF
CTACK	Dtk	EGFR	ENA-78	Fas	FGF4	FGF9	CSF3	GITRL	GITR	GRO	GRO-α	HCC-4	HGF
ICAM1	ICAM3	IGFBP3	IGFBP6	sIGF-1R	IL1R4	IL-1 R1	IL-11	IL-12 p40	IL-12 p70	IL-17	IL-2 Rα	sIL-6R	IL-8
ICAM1	ICAM3	IGFBP3	IGFBP6	sIGF-1R	IL1R4	IL-1 R1	IL-11	IL-12 p40	IL-12 p70	IL-17	IL-2 Rα	sIL-6R	IL-8
I-TAC	XCL1	MIF	MIP-1α	MIP-1β	MIP-3β	MSP-α	NT-4	OPG	OSM	PLGF	sgp130	sTNFR2	sTNFR1
I-TAC	XCL1	MIF	MIP-1α	MIP-1β	MIP-3β	MSP-α	NT-4	OPG	OSM	PLGF	sgp130	sTNFR2	sTNFR1
TECK	TIMP1	TIMP2	THPO	TRAILR3	TRAILR4	μPAR	VEGF-A	VEGF-D	NEG	NEG	NEG	NEG	NEG
TECK	TIMP1	TIMP2	THPO	TRAILR3	TRAILR4	μPAR	VEGF-A	VEGF-D	NEG	NEG	NEG	NEG	NEG

The markers of CV-MSCs derived from three healthy donors were evaluated by LSRII flow cytometer (BD). Data was analyzed by Flowjo7.6 software.

Table S3: Phenotype of three donors derived CV-MSC.

Markers (%)	CV-MSC Donor 1	CV-MSC Donor 2	CV-MSC Donor 3	Mean ±SEM
CD14	4.43	0.05	5.53	3.33 ±1.6
CD29	99.82	99.85	99.40	99.69 ±0.14
CD31	0.02	0.24	0.93	0.39 ±0.27
CD45	0.45	0.79	2.24	1.16 ±0.54
CD54	65.24	84.07	93.30	80.87 ±8.25
CD73	99.14	99.32	99.20	99.22 ±0.05
CD90	99.86	99.85	94.10	97.94 ±1.91
CD105	99.90	99.04	95.70	98.21 ±1.28
VCAM-1/CD106	73.02	54.77	60.90	62.9 ±5.36
CD144	0.21	0.05	5.47	1.91 ±1.78
CD133	0.21	0.06	0.08	0.11 ±0.04
CD166	48.40	72.55	94.20	71.72 ±13.23
HLA-ABC	91.10	98.24	93.61	94.32 ±2.09
HLA-DR	0.12	3.09	0.64	1.28 ±0.91

Cytokine profiles of VCAM-1$^+$ and VCAM-1$^-$ CV-MSCs revealed 15 factors angiogenic cytokines to be significantly different. Among them, 13 cytokines showed an increased expression in VCAM-1$^+$ CV-MSC in comparison with VCAM-1$^-$CV-MSC.

Table S4: Differential angiogenesis cytokines of VCAM-1$^-$ and VCAM-1$^+$ CV-MSC.

Angiogenic Cytokines	VCAM-1$^-$CV-MSC Mean	VCAM-1$^+$ CV-MSC Mean	Ratio
Angiogenin	2482	3308	1.333
GM-CSF/CSF2	1348	7721	5.729
IL-1α	506	814	1.609
IL-1β 45	155	1021	6.572
MCP-3/CCL7	6238	8224	1.318
RANTES/CCL5	986	675	0.685
TARC	310	230	0.743
Angiopoietin-2	219	296	1.353
CTACK/CCL27	161	211	1.308
GCSF/CSF3	571	1161	2.036
GRO-alpha/CXCL1	399	593	1.487
HGF	11110	14751	1.328
IL-8	610	1041	1.706
Osteoprotegerin/OPG	13636	19917	1.461
uPAR	282	477	1.691

Supplemental Fig.1: sVCAM-1 concentration in 48hours CM of VCAM-1^{+} and VCAM-1^{-}CV-MSCs were measured by ELISA. Both of them secreted very fewer sVCAM-1 in normal conditions (<20pg/ml). Additionally, there is no statistically difference between VCAM-1^{+} and VCAM-1^{-} CV-MSCs in sVCAM-1 secretion (p>0.05).

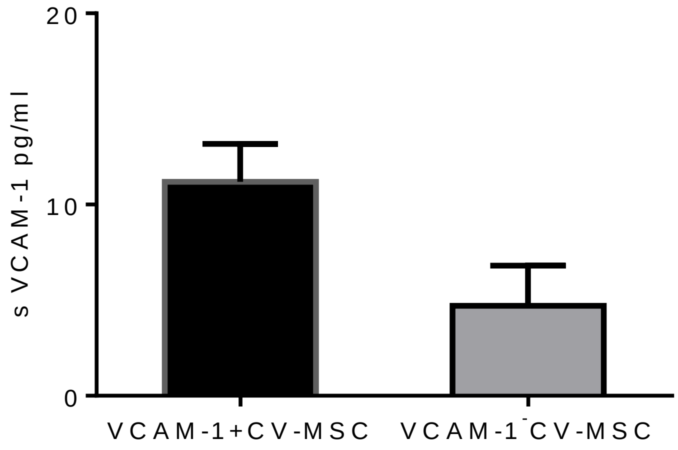

Supplemental Fig.2: The endothelial-like cells derived from VCAM-1$^+$ and VCAM-1$^-$ CV-MSCs were harvested after *in vitro* endothelial-induction and immunostained by anti-vWF antibodies to evaluate their endothelial-differentiation capacities. Photos were captured by using Confocal Microscope (Leica, scale bar=40μm). Cells labeled with the second antibodies were used as control. Endothelial-like cells induced from VCAM-1$^+$ (*b*) and VCAM-1$^-$CV-MSCs (*c*) were stained with anti-vWF antibodies, respectively. No apparent differences were seen between VCAM-1$^+$ and VCAM-1$^-$ CV-MSCs.

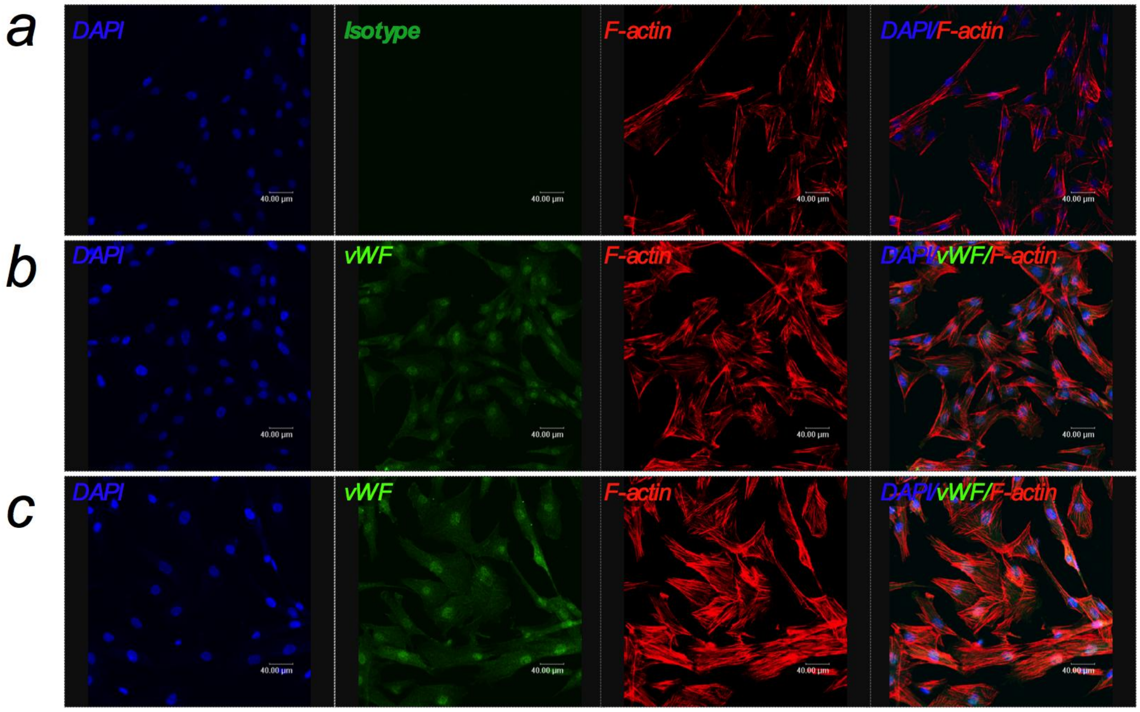

Supplement: Additional file 1: — is Table S1 presenting primers for real-time reverse transcription PCR, Table S2 presenting the description of the human cytokine antibody array (AAH-CYT-G1000), Table S3 presenting the phenotype of three donor-derived CV-MSCs, Table S4 presenting differential angiogenesis cytokines of VCAM-1–CV-MSCs and VCAM-1+CV-MSCs, Fig. S1 showing sVCAM-1 concentration in 48-hour CM of VCAM-1+CV-MSCs and VCAM-1–CV-MSCs measured by ELISA, and Fig. S2 showing the endothelial-like cells derived from VCAM-1+CV-MSCs and VCAM-1–CV-MSCs harvested after in vitro endothelial induction and immunostained by anti-vWF antibodies to evaluate their endothelial differentiation capacities. (PDF 524 kb) [file 13287_2016_297_MOESM1_ESM.pdf]
